# Assessment of *Toxoplasma* Seropositivity in Children Suffering from Anxiety Disorders

**Published:** 2017

**Authors:** Shahla AFSHARPAIMAN, Mohammad Hossein KHOSRAVI, Mojtaba MAHMOODINEJAD, Shahnaz SHIRBAZOO, Susan AMIRSALARI, Mohammad Torkaman, Shokoofeh Radfar

**Affiliations:** 1 Health Research Center, Baqiyatallah University of Medical Sciences, Tehran, Iran.; 2 Student Research Committee, Baqiyatallah University of Medical Sciences, Tehran, Iran.; 3 Department of Parasitology, Baqiyatallah University of Medical Sciences, Tehran, Iran.; 4 New Hearing Technologies Research Center, Baqiyatallah University of Medical Sciences, Tehran, Iran.; 5 Department of Pediatrics, Baqiyatallah University of Medical Sciences, Tehran, Iran.; 6 Psychiatry Department, Baqiyatallah University of Medical Sciences, Tehran, Iran.

**Keywords:** Anxiety disorders, Toxoplasmosis, *Toxoplasma gondii*, Antibodies

## Abstract

**Objective:**

This study aimed to assess the seroprevalence of *Toxoplasma gondii* in children with anxiety disorders.

**Materials & Methods:**

This cross-sectional study was conducted between Sep 2012 and May 2013 in Pediatrics Clinic of Baqiyatallah Hospital, Tehran, Iran. Children were assessed clinically. Diagnosis of patients with anxiety disorders was based on DSM-4 system, performed by child psychiatrist. Then their anti-*Toxoplasma* antibodies were measured. A questionnaire was verbally administered to all individuals’ parents including demographic information and questions about life style, family history, medical history, economic situation, residence, nutritional patterns and contact with animals.

**Results:**

Ninety-six male and female cases with a mean age of 8.56±2.5 and 8.42±1.9 yr underwent analysis. Anti-* T. gondii* IgG antibody was found in one case of each group. There was no significant difference between case and control groups for serum *Toxoplasma *IgG antibody (*P*=0.14). No case individuals had Anti-* T. gondii* IgM antibody, while it was found in one control individual. No significant difference was seen between case and control groups for* Toxoplasma *IgM antibody (*P*=0.27).

**Conclusion:**

Toxoplasmosis has no direct effect on the incidence of anxiety disorders. More studies are needed with a larger volume of individuals in future.

## Introduction

Toxoplasmosis is a public health problem worldwide, caused by *Toxoplasma gondii, *an obligate intracellular protozoan. Toxoplasmosis may involve various organs including central nervous system considered to result in anxiety disorders, defined as a group of mental disorders identified by feelings of fear and anxiety about future events (1), like simple phobia, social phobia, agoraphobia, generalized anxiety disorders (GAD) and obsessive-compulsive disorder (OCD), while the infection in the early childhood may cause neurodegenerative disease. 

There is a hypothesis suggesting that the metabolic products released from the parasite cyst in the brain cause inflammation and encephalitis with an associated alteration of behavior (2). “There was also an evidence of focal inflammation with disrupted tissue cysts in mice (3). *Toxoplasma*-infected mice are reported to have deficits in learning capacity and memory” (4). The neurophysiological mechanisms of these changes may be related to increased concentrations of dopamine observed in the brains of chronically infected mice (5). “Antiparasitic agents, as well as anti-psychotics, are effective in treating parasitosis” (6). The toxoplasmosis has no direct effect on the risk of schizophrenia and is just an indication of previous contacts with cat (7). Therefore, the effect of toxoplasmosis on behavior has remained as controversy.

This study aimed to assess the antibodies for toxoplasmosis in children suffering from anxiety disorders and healthy controls to discuss the association between toxoplasmosis and the etiopathogenesis of anxiety disorders.

## Materials and Methods

This cross-sectional study was conducted between Sep 2012 and May 2013 in Pediatrics Clinic of Baqiyatallah Hospital, Tehran, Iran. Considering the three-percent prevalence of positive IgG for *T. gondii* in general population and α=0.05, a sample volume of 45 was calculated for each group. Children referred to Child Psychiatry Clinic of our hospital were assessed clinically. At first, all the individuals were assessed by both pediatrician and child psychiatrist for signs of anxiety disorders. Children with signs of anxiety disorder were underwent clinical assessments. Diagnosis of these patients was based on DSM-4system, performed by child psychiatrist. Children with underlying disorders similar to mental disorders were excluded from the study. Then they were referred to laboratory in order to have their anti-*Toxoplasma* antibodies measured by ELISA test. Five ml of blood samples were taken from each individual under sterile conditions. The blood samples were centrifuged at 1000 rpm and the sera stored at –70 ^o^C until analyzed. A commercial Micro Enzyme Immuno Assay (mEIA) kit (Diasorin S.P.A. Via Crescentino, snc 13040 Saluggia (VC), Italy) was used for the detection of anti-*Toxoplasma *IgG and IgM antibodies.

A questionnaire was verbally administered to all individuals’ parents including information about their age, education, life style, family history, medical history including receiving any blood products, economic situation, residence, nutritional patterns, and contact with animals.

The study was approved by Ethics Committee of Baqiyatallah University of Medical Sciences. The aims of the study were explained to all parents and they were asked to sign an informed consent form. This study had no disturbances on patients’ treatment process. All the personal information was kept anonymous. Individuals were allowed to leave the study at the time of intention.

Quantitative variables are presented as mean ± standard deviation (SD). Qualitative variables are presented as frequencies (percentages) compared using the chi-square test. The univariate data analysis was done using the contingency tables. A logistic regression method was used for the multivariate analysis. All statistical tests were two-sided and a *P* value of less than 0.05 was considered statistically significant. Statistical analysis was performed using SPSS 16.0 software for windows (SPSS Inc. Chicago, IL).

## Results

Eventually, 96 male and female children, categorized as case and control groups, underwent analysis. Case and control groups, with 23 male and 25 female individuals each, had a mean age of 8.56±2.5 and 8.42±1.9, respectively (Table 1). All children were residents of Tehran and had no history of receiving any blood products. There was no significant difference between both groups for age (*P*=0.22). All individuals in case group had a kind of anxiety disorders such as OCD or phobias while control individuals had none of them. Case and control individuals had a mean birth weight of 3008.23±807.83 and 3108.42±847.67 gr (Table 2), respectively which was not significantly different between the groups (*P*=0.17).

**Table 1:** Demographical Properties of Case Group and Control Group

**Demographical properties**	**Case group**	**Control group**	***P*** ** Value**
**Gender, male N(%)**	23(47.9)	23 (47.9)	0.62
**Age**, year (mean±SD)	8.56 ± 2.5	8.42 ± 1.9	0.22
**Disease Duration**, month (mean±SD)	35 ± 23.8	-	-
**Economic Situation**			
**High N(%)**	4 (8.3)	5 (10.4)	
**Middle N(%)**	42 (87.5)	41 (85.4)	
**Low N(%)**	2 (4.2)	2 (4.2)	
**Weight, **Kg (mean±SD)	29 ± 5.3	28± 10.6	0.36

**Table 2:** Assessed Factors Considered to be Probably Related With Disease

**Factors **	**Case Group**	**Control Group**	**p Value**
**Birth Age**			
Term Birth N(%)	40 (83.4)	44 (91.7)	0.11
Preterm birth N(%)	8 (16.6)	4(8.3)	
**Birth Weight, gr **(mean±SD)	3008.23 ± 807.83	3108.42±847.67	0.17
**Mother’s Infectious Diseases During Pregnancy **			
Negative N(%)	41 (85.4)	47 (97.9)	0.22
Positive N(%)	7 (14.6)	1 (2.1)	
**Medicinal Therapy**			
**Male N(%)**	14 (29.2)		
**Female N(%)**	18 (37.5)		
**Toxoplasmosis-Like Symptoms in Past**			
Negative N(%)	47 (97.9)	47 (97.9)	0.32
Positive N(%)	1 (2.1)	1 (2.1)	
**Psychiatric Diseases in Relatives **			
Negative N(%)	25 (52.1)	43 (89.6)	0.02
Positive N(%)	23 (47.9)	5 (10.4)	
**Undercooked Meats Consumption**			
Negative N(%)	48 (100)	47 (97.9)	0.32
Positive N(%)	0	1 (2.1)	
**Raw Vegetables Consumption**			
Negative N(%)	19 (39.6)	18 (37.5)	0.5
Positive N(%)	29 (60.4)	30 (62.5)	
**History** **of close contact with animal**			
Negative N(%)	45 (93.7)	46 (95.8)	0.65
Positive N(%)	3 (6.3)	2 (4.2)	
**Water Source**			
Unrefined N(%)	1 (2.1)	0	
Refined N(%)	47 (97.9)	48 (100)	

Anti-*T. gondii* IgG antibody was found in one case of each group. The individual with anti-*T. gondii* IgG in the case group also had OCD at the same time. There was no significant difference between case and control groups for serum *Toxoplasma *IgG antibody (*P*=0.14). No case individuals had Anti-*T. gondii* IgM antibody, while it was found in one control individual. No significant difference was seen between case and control groups for* Toxoplasma *IgM antibody (*P*=0.27).

In the case group, 40 patients (83.4%) and in control group 44 individuals (91.7%) had term birth, while 8 patients (16.6%) from case group and 4 individuals (8.3%) in control group had preterm birth (*P*=0.11). There was no significant difference between case and control groups for birth age. 

Forty-one mothers (85.4 %) in case and 47 (97.9%) in control individuals had no symptoms compatible with toxoplasmosis or other viral or bacterial infectious diseases during pregnancy. No significant difference was seen between study individuals for mother’s infectious disease during pregnancy. Only one case individual (2.1%) had toxoplasmosis-like symptoms in past just like the control group. There was no significant difference between case and control individuals for toxoplasmosis-like symptoms in past (*P*=0.32).

History of raw vegetable consumption was positive in 29 (60.4%) case and 30 (62.5%) control individuals. There was no significant association between anxiety disorders and raw vegetable consumption (*P*=0.5). In case group 23 (47.9%) and in control group 5 (10.4%) individuals had a positive family history for anxiety disorders. There was a significant difference between case and control individuals for this factor (*P*=0.02).

Three (6.3%) case and 2 (4.2%) control individuals had a history of close contact with animals. Cats and dogs were excluded because of low ownership among study individuals. There was no significant difference between study individuals for close contact with animals (*P*=0.65). 

## Discussion

There was no significant difference for anti-*Toxoplasma* antibodies between case and control group members. Therefore, seropositivity of anti-toxoplasma antibodies is not associated with incidence of anxiety disorders.

OCD is an anxiety disorder and the etiology of OCD is unknown thought. Family genetic data show that the familial forms of OCD may be related with genetic specificities (8). The present study showed that there was an association between positive familial history and anxiety disorders. Previous studies have shown a relationship between infectious diseases and OCD symptoms progression (9) and that latent infection of *T. gondii* has effects on behavior and learning capacity. Exprimental infection in mice decreases learning function, memory, response to prolonged stimuli and overall activities (10) and that *T. gondii* infection may effect on animal response to environmental stimuli and can even be effective in the processes within the brain. Acquired toxoplasmosis can cause neurological symptoms or can mimic neurological and psychiatric syndromes.

Suicide attempt was highly associated with anti-*Toxoplasma* IgG antibody serum level (11). In our study, no significant difference was found between case and control groups regarding serum anti-*Toxoplasma* antibodies. In addition, no significant difference was seen for anti-*Toxoplasma* antibodies among patients with anxiety disorders and OCD too and there was only one IgG seropositive OCD patient. 

OCD symptoms intensity is related to higher levels of anti-*Toxoplasma* antibodies in serum and anti-protozoan treatment causes a decrease in antibody levels, which looks like a confirmation of neurotoxoplasmosis (12). In another study, anti-protozoan medications decreased *Toxoplasma* antibodies in two children with toxoplasmosis and OCD according to DSM-IV and DSM-III-R tests and made OCD completely cured (13). This finding shows a cause and effect relationship between acquired toxoplasmosis and OCD which is not in agreement with our study except the decreasing role of anti-*Toxoplasma* medications in OCD symptoms. Schizophrenia was associated with higher percentages of anti-*Toxoplasma* antibodies (14, 15). There was a significant relationship between OCD and female gender as well as older ages (16). In a study on 12-yr-old, individuals have concluded that preterm children had a significantly lower learning power, more anxiety disorders and needed more education comparing to control group (17). In a study on 11-yr old children with low birth weight, they had more anxiety disorders than children with normal birth weight (18). While in the present study there was no significant difference between case and control groups for this parameter; however, our study aimed to assess the relation between toxoplasmosis seropositivity and anxiety disorders in children so we cannot completely compare these parameters with our results.

Latent toxoplasmosis may play different roles in etiopathogenesis of neuropsychiatric disorders and that psychiatric features are common in parasitic infections like toxoplasmosis (19, 20).

*Toxoplasma* infection was highly associated with generalized anxiety disorders and might play a role in their development (21).

Low sample volume and participating of young cases could have effects to some extent on the results of the present study as well as self-reported data obtained from questionnaires, which could potentially introduce a bias.

**In Conclusion, **serum anti-*Toxoplasma* antibodies are not associated with incidence of anxiety disorders in low *Toxoplasma* seroprevalence children. We suggest future studies with a larger sample volume and a different study design to compare positive cases of anti-*Toxoplasma* antibodies to negative cases with anxiety disorders in different age groups.
